# High Healthcare Utilization in Adolescents with Sickle Cell Disease Prior to Transition to Adult Care: A Retrospective Study

**DOI:** 10.36469/10512

**Published:** 2019-10-09

**Authors:** Julie Kanter, Menaka Bhor, Xin Li, Frank Li, Jincy Paulose

**Affiliations:** 1 University of Alabama at Birmingham, Division of Hematology and Oncology; 2 U.S. Oncology Health Economics and Outcomes Research Novartis Pharmaceuticals Corporation; 3 Health Economics and Outcome Research KMK Consulting Inc.; 4 U.S. Oncology Medical Novartis Pharmaceuticals Corporation

**Keywords:** claims database, treatment patterns, pain crises, healthcare utilization, adolescents, sickle cell disease

## Abstract

**Background:** The transition from pediatric to adult care in the US is often difficult for individuals with sickle cell disease (SCD). Young adults (18 to 25 years of age) have higher acute care utilization and an increased risk of poor outcomes. The current study was designed to provide greater insights into patients (16 to 18 years of age) with SCD prior to their transition to adult care.

**Objectives:** To describe current treatment patterns, pain crises prevalence, SCD-related complications, and healthcare resource utilization (HCRU) in 16-to-18-year-old patients with SCD.

**Methods:** From 1/1/2015-6/30/2017 using MarketScan Databases, patients were included if they were 16 to 18 years old at the index date, had ≥1 inpatient or 2 outpatient SCD diagnosis claims during the identification period, and were continuously enrolled in the database for at least one year prior (baseline) and post index date. Outcomes included medications, disease management interventions, Charlson Comorbidity Index (CCI), vaso-occlusive (VOC) crises requiring healthcare visits, HCRU, and SCD complications.

**Results:** 1,186 patients were included; most (64.3%) were female. The mean CCI was 1.3 (SD: 0.7). In the overall cohort, patients experienced an average of 3.9 (SD: 4.2) VOCs and most patients (61.1%, n=725) had chronic complications during the one-year follow-up. Pulmonary disease (31.1%, n=369) was the most frequent complication; blood transfusions (mean: 8.4 [SD:7.0]) and iron-chelating therapies (mean: 8.6 [SD:10]) were the most common interventions and medications, respectively. In the 16 to 18 year old group, patients with SCD had 2.0 (SD = 2.3) hospital admissions, 3.4 (SD = 4.0) ER visits, and 5.0 (SD = 4.2) office visits.

**Conclusions:** Prior to transition to adult care, adolescents with SCD already have significant acute and chronic disease-related complications, possibly contributing to frequent healthcare visits. Increased attention to this age group, including improvements in disease modifying therapy, are needed prior to transition to adult care systems to improve outcomes.

## Introduction

Sickle cell disease (SCD) is an inherited disorder characterized by a defect in the gene for hemoglobin.^1^ In SCD, the activation of endothelial cells initiate adhesive interactions with red blood cells, white blood cells, and platelets due to chronic vascular damage.^2^ The chronic inflammatory environment within blood vessels leads to increased expression of adhesion mediators resulting in multicellular adhesion.^3^ Pain from acute vaso-occlusion in SCD is the hallmark of the disease caused (in part) by erythrocytes and leukocytes that become trapped in the microcirculation resulting in vascular obstruction and tissue ischemia.^4^ These painful events, termed vaso-occlusive crisis (VOC), require acute care in a hospital and can adversely affect an individual’s quality of life (QoL).^2^ Ischemic damage from ongoing vaso-occlusion can lead to organ failure.^2^

SCD most commonly affects people of African, Mediterranean, and Asian heritage.^1,5^ The prevalence of SCD in the United States (US) is estimated at nearly 100 000, and 95% of SCD cases occur in people having African American heritage.^6,7^ About 2 000 babies are born with SCD in the US every year.^8^ A California and Illinois study in the early nineties showed the cumulative mortality rate was 1.5/100 African-American child with SCD.^9^

Adolescent and young adult patients with SCD experience a wide array of acute and chronic complications,^10–13^ with pain or intensive painful episodes (VOC), accounting for the vast majority of these complications.^10^ Kanter et al. (2018) showed that 12-to-18-year-old patients with SCD experienced more frequent pain than their 7-to-12-year-old counterparts.^12^ These findings are concerning when reviewing available mortality data in patients <18 years of age. Currently available data from the Dallas Newborn Cohort (DNC) suggest patients aged 16 to 18 years have a higher death rate (2.91/100 patient-years) compared with younger patients.^14^ Another DNC study from 2010 corroborated this and found that the highest incidence of death was in 15-to-20-year-old patients (at least 3 times the incidence in other younger age groups).^15^

About 10% of children with SCD have a symptomatic stroke during childhood.^16–19^ Ischemic strokes account for most of the overt strokes in children with SCD, while hemorrhagic strokes tend to become more frequent in young adulthood.^13^ A study conducted in east London found the estimated risk of stroke at 4.3% for children aged 15 years with SCD.^20^

The transition from pediatric to adult care in the US is often difficult for individuals with SCD. Multiple articles report that young adults (18 to 25 years of age) have higher acute care utilization and an increased risk of poor outcomes.^10,11,13–15,21,22^ Published data are limited on acute and chronic complications, specifically associated with SCD in children *prior to the time of transition* to adult care.^13,20,23–27^ More information is needed about healthcare resource utilization (HCRU) and treatment patterns in young patients with SCD before they transition to adult care.^15,21,28–32^ Improved understanding of the HCRU in adolescents may help predict individuals at higher risk post-transition.

The current study was designed to provide greater insights into patients with SCD prior to their transition to adult care. In this study, we sought to describe within the 16-to-18-year-old age group current treatment patterns, the prevalence of pain crises (VOCs), HCRU, and SCD-related complications to understand the management of SCD.

## Materials and Methods

### Data Source

A retrospective observational claims analysis was conducted using data from the Truven MarketScan® Medicaid Research Databases.^33^ Healthcare data are provided under a variety of fee-for-service (FFS), point-of-service (POS), or capitated health plans. Types of health plans included health maintenance organization (HMO) and POS or comprehensive plans. Detailed cost, use, and outcomes data for healthcare services performed in both inpatient and outpatient settings were collected.^33^

Data for the current study were de-identified according to the US Health Insurance Portability and Accountability Act and managed according to MarketScan customer data usage agreements. The study did not involve the collection, use, or transmission of individually identifiable data thus Institutional Review Board approval was not required.

### Subject Selection and Patient Population

Patients with SCD diagnoses were identified using the International Classification of Diseases (ICD)-9-Clinical Modification (CM) and ICD-10-CM diagnosis codes from January 1, 2015 through June 30, 2017 (Figure 1–Patient Selection; Appendix B). The first SCD identification (claim) date in the identification period was considered the index date. Patients were then assigned into the 16-to-18-year-old age group based upon their age at the first SCD claim and were traced back 12 months prior to the index (baseline period) and followed for 12 months post index (follow-up period).

Patients were included if they had at least one inpatient claim or two outpatient claims with SCD diagnoses during the identification period from January 1, 2015 through June 30, 2017. In addition, they had to have continuous enrollment with medical and pharmacy benefits for at least 12 months pre-index and 12 months post-index and were between 16 and 18 years of age at the index date.

**Figure A1.**
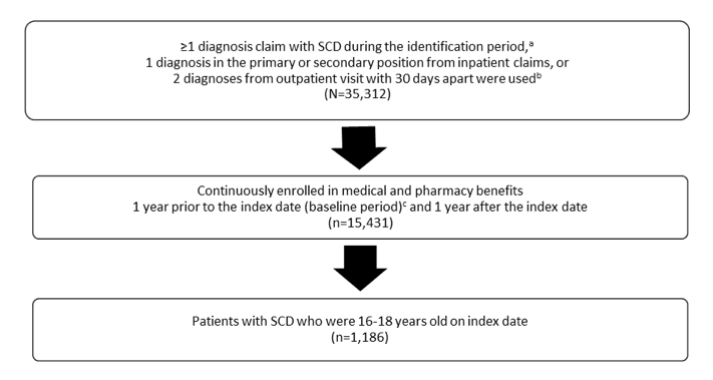
Diagram of SCD^a^ Patient Selection Flow ^a^SCD, sickle cell disease. ^b^Identification period was between January 1, 2015 to June 30, 2017. ^c^First diagnosis served as index date. ^d^Baseline period: 1 year prior to the index date.

### Demographic Variables

The age of the subject at the index date was recorded. Other demographic variables included gender and race/ethnicity. Clinical information at baseline consisted of the Charlson Comorbidity Index (CCI). A score of zero indicates that no comorbidities were found, while the higher the score, the more likely the outcome of interest will result in higher resource use or mortality.^34^

### Outcome Measures

Outcome measures encompassed SCD-related HCRU (a listing of ICD codes is provided in Appendix E) in the one-year follow-up period including: length of stay (LOS) for inpatient hospitalization (count of hospital days); hospital admissions (count of number of SCD-related hospitalization); emergency room (ER) visits (count of number of SCD-related ER visits); office visits (count of number of SCD-related office visits); and other outpatient visits (count of number of SCD-related other outpatient visits).

### Treatment Patterns

SCD treatment patterns (see Appendix C, Appendix D, and Appendix E) were measured according to a frequency distribution of the treatment type while SCD management was assessed as a frequency distribution of the SCD reported procedure type. VOC episodes were analyzed as counts of crises (based on ICD-9/10 designation) in the 1-year follow-up period, overall, and per patient. We defined a VOC event as any continuous event that lasted up to 9 days (allowing a 3-day gap; in other words, if a patient had another pain crisis related service within a 3-day window of the first crisis, we still counted it as one event, not as two events), based on expert opinion. The number of VOC episodes were recorded in the follow-up period for the overall population and for subgroups of patients based on the amounts of annual crises (0, 1, and ≥2). Individual SCD complication rates in the one-year follow-up period were recorded for the overall population and for the subgroups based on the numbers of annual crises. Complications were divided into acute and chronic and according to organs/systems affected (e.g., cerebrovascular, hepatic, pulmonary, renal, spleen, other; Appendix F) as per ICD-9/10 data. Medications were defined by categories: antibiotics, nonsteroidal anti-inflammatory drugs (NSAIDS), antimetabolites (only hydroxyurea), chelating medications and opioid medications (Appendix E).

### Statistical Analysis

All variables were analyzed descriptively. Numbers and percentages are provided for categorical variables. Means and standard deviation (SD) are provided for continuous variable and evaluated each time for a subset of patient who had at the given intervention or medication and a healthcare facility visit. All analyses were performed on an analytical platform consisting of SAS version 9.4 (SAS Institute Inc., Cary, NC) and data management software.

## Results

### Overall Patient Cohort During the 1-Year Follow-up

The average age of the 1186 patients was 17.0 years with females representing 64.3% (n=763) of the overall patient cohort (Table A1). The majority (76.0%, n=901) of patients were African American, which was as expected based on published prevalence data.^6,7^ The mean CCI score for patients with CCI data was 1.3 (SD = 0.7). Patients with at least one VOC experienced an average of 3.9 (SD = 4.2) VOCs requiring healthcare visits per year. Approximately 1 in 5 (17.4%, n = 206) patients had acute complications while over half (61.1%, n = 725) were affected by chronic complications from SCD. Of the complications identified, those relating to impaired pulmonary function (including acute chest syndrome [ACS]) were the most prevalent (31.1% of the total, n = 369). The mean hospital LOS for patients with at least one hospital visit was 9.0 (SD = 14.4) days. The two highest SCD-related HCRUs were attributed to non-acute visits either office visits (mean = 5.0, SD = 4.2) and other outpatient visits (mean = 7.9, SD = 11.0). Overall, the highest claims for SCD management was for blood transfusions (mean = 8.4, SD = 7.0). For medications, the highest numbers of prescription claims were associated with iron chelating therapies (mean = 8.6, SD = 10.0) and hydroxyurea (mean = 5.3, SD = 3.2.)

**Table A1.** Demographics, Disease Characteristics, HCRU, and Medications for SCD Patients 16-18 Years of Age Based on the Number of Vaso-occlusive Crises.

	**Overall 16-18-year old (N=1186)**	**16-to-18-year-old**		
		**Number of Vaso-occlusive Crises**		
		**0** **(n=716)**	**1 (n=137)**	**2+ (n=333)**
**Patients with SCD, n (%)**		60.4	11.6	28.1
**Age, mean (SD)**	17.0 (0.8)	17.1 (0.8)	16.9 (0.8)	17.0 (0.8)
**Gender, n (%)**				
**Male**	423 (35.7)	199 (27.8)	65.0 (47.4)	159 (47.7)
**Female**	763 (64.3)	517 (72.2)	72 (52.6)	174 (52.3)
**Ethnicity, n (%)**				
**White**	32 (2.7)	26 (3.6)	2 (1.5)	4 (1.2)
**Black**	901 (76.0)	587 (82.0)	101 (73.7)	213 (64.0)
**Hispanic**	14 (1.2)	12 (1.7)	0 (0)	2 (0.6)
**Other**	233 (19.6)	85 (11.9)	34 (24.8)	114 (34.2)
**Missing/Unknown**	6 (0.5)	6 (0.8)	0 (0)	0 (0)
**Charlson Comorbidity Index, mean (SD)**	1.3 (0.7), n=369	1.3 (0.8), n=187	1.3 (0.5), n=41	1.4 (0.8), n=141
**VOC Episode, mean (SD)**	3.9 (4.2), n=470	0 (0), n=0	1.0 (0), n=137	5.1 (4.4), n=333
**SCD Complications, n (%)**				
**Acute**	206 (17.4)	70 (9.8)	22 (16.1)	114 (34.2)
**Chronic**	725 (61.1)	379 (52.9)	80 (58.4)	266 (79.9)
**Cerebrovascular Disease**	69 (5.8)	45 (6.3)	5 (3.6)	19 (5.7)
**Hepatic Disease**	76 (6.4)	21 (2.9)	11 (8.0)	44 (13.2)
**Pulmonary Disease**	369 (31.1)	169 (23.6)	37 (27.0)	163 (48.9)
**Renal Disease**	106 (8.9)	55 (7.7)	10 (7.3)	41 (12.3)
**Spleen Disease**	6 (0.5)	0 (0)	0 (0)	6 (1.8)
**Other**	576 (48.6)	275 (38.4)	60 (43.8)	241 (72.4)
**SCD-related HCRU, mean (SD)**				
**Length of Stay (Hospital)**	9.0 (14.4), n=544	4.2 (8.1), n=248	5.4 (10.1), n=41	14.3 (17.6), n=255
**Hospital Admissions**	2.0 (2.3), n=544	1.1 (0.4), n=248	1.3 (0.8), n=41	3.0 (3.0), n=255
**ER Visits**	3.4 (4.0), n=687	1.8 (1.1), n=283	1.9 (1.3), n=94	5.3 (5.3), n=310
**Office Visits**	5.0 (4.2), n=806	4.1 (4.0), n=357	4.7 (3.4), n=130	6.1 (4.4), n=319
**Other Outpatients Visits**	7.9 (11.0), n=831	5.6 (9.9), n=384	6.7 (7.7), n=128	11.1 (12.5), n=319
**SCD Managements, mean (SD)**				
**Blood transfusions**	8.4 (7.0), n=137	9.0 (7.3), n=50	8.1 (4.9), n=15	8.0 (7.1), n=72
**Pneumococcal vaccines**	1.1 (0.3), n=100	1.1 (0.3), n=30	1.1 (0.3), n=25	1.2 (0.4), n=45
**Meningococcal vaccines**	1.2 (0.5), n=227	1.2 (0.5), n=112	1.2 (0.5), n=30	1.2 (0.5), n=85
**Bone marrow transplants**	1.0 (0.0), n=2	1.0 (0.0), n=2	0.0 (0.0), n=0	0.0 (0.0), n=0
**Transcranial doppler ultrasonography**	1.2 (0.5), n=105	1.3 (0.7), n=28	1.1 (0.3), n=22	1.2 (0.5), n=55
**SCD Medications, mean (SD)**				
**Antibiotics**	2.7 (2.6), n=691	2.4 (2.2), n=387	2.9 (2.7), n=69	3.2 (3.0), n=235
**Antidepressants**	3.9 (4.1), n=137	3.0 (2.9), n=76	5.6 (3.5), n=10	4.9 (5.2), n=51
**Antimetabolite**	5.3 (3.2), n=272	5.9 (3.5), n=48	5.8 (3.2), n=51	5.0 (3.1), n=173
**Iron chelating therapy**	8.6 (10.0), n=70	10.2 (12.3), n=29	4.3 (2.7), n=9	8.3 (8.7), n=32
**NSAIDs**	2.9 (2.5), n=742	2.1 (1.9), n=370	2.7 (2.1), n=100	4.1 (2.9), n=272
**Opioids**	4.7 (5.9), n=672	2.0 (2.6), n=263	3.5 (3.2), n=101	7.4 (7.3), m=308
**Others**	4.3 (3.2), n=353	4.4 (3.4), n=86	4.7 (3.3), n=69	4.1 (3.0), n=198

ER, emergency room; HCRU, healthcare resource utilization; NSAID, Nonsteroidal anti-inflammatory drug; SCD, sickle cell disease; SD, standard deviation; VOC, vaso-occlusive.

### Subgroup Analysis of Patients with 0, 1, and ≥2 Vaso-occlusive Episodes

#### Subgroup 1: Patients without any VOC in the 1-Year Follow-up

There were 716 patients (60.4%) in the cohort who had no VOCs requiring healthcare visits (see Table A1). The majority (n = 517, 72.2%) of the patients not suffering from VOCs were females. Only about 1 in 10 (9.8%, n = 70) patients in this subgroup had acute complications while over half of them (52.9%, n = 379) experienced chronic complications. Chronic pulmonary disease (see Appendix F) was the most prevalent complication identified (23.6%, n = 169). Amongst those who had at least one hospital visit (not for VOC per ICD-9/10 codes), the average hospital LOS was 4.2 (SD = 8.1) days. In this same sub-group, the two highest SCD-related HCRUs were for office visits (mean = 4.1, SD = 4.0) and other outpatient visits (mean = 5.6, SD = 9.9). In the subset of patients, the most common intervention was blood transfusions (mean = 9.0, SD = 7.3). Similar to the overall patient cohort, the highest claims for medications/treatments in this subset were for iron-chelating therapies (mean = 10.2, SD = 12.3) and hydroxyurea (5.9, SD = 3.5). NSAID claims were comparable at 2.1 (SD = 1.9) to opioid use (2.0, SD = 2.6) for this group.

#### Subgroup 2: Patients with 1 VOC in the 1-Year Follow-up

The subgroup with 1 VOC requiring healthcare visits comprised of 137 (11.6% of total) patients. A substantial proportion of patients with 1 VOC experienced both acute (16.1%, n = 22) and chronic complications (58.4%, n = 80). Chronic pulmonary disease was comparable to the group without VOCs (27.0%, n = 37). The average hospital LOS for this subgroup was 5.4 days (SD = 10.1 days) including SCD-related hospitalizations. The two highest SCD-related HCRUs were office visits (mean = 4.7, SD = 3.4) and other outpatient visits (mean = 6.7, SD = 7.7). The highest SCD-related HCRU was for blood transfusions (mean = 8.1, SD = 4.9). The highest medication claims were for hydroxyurea (mean = 5.8, SD = 3.2). Opioid claims in this group were 3.5 (SD = 3.2) (slightly higher than the previous group without VOC events). As with the group without VOC-related hospitalizations, NSAID claims were comparable at 2.7 (SD = 2.1) to opioid use (3.5, SD = 3.2) for this group.

#### Subgroup 3: Patients with ≥ 2 VOC in the 1-Year Follow-up

The third subgroup of patients had ≥2 VOC resulting in hospital utilization. This group included 333 patients (28.1%) and had an average of 5.1 (SD = 4.4) pain episodes. About 1 in 3 of these patients (34.2%, n = 114) had acute complications and the vast majority (79.9%, n = 266) had chronic complications. About half (48.9%, n = 163) of the patients in this group experienced pulmonary complications. The average LOS was longer in this group approximately 14.3 days (SD:17.6). HCRUs were high for this group of patients: hospital admissions (mean = 3.0, SD = 3.0), ER visits (mean = 5.3, SD = 5.3), office visits (mean = 6.1, SD = 4.4), and other outpatient visits (mean = 11.1, SD = 12.5). Blood transfusions were frequent (mean = 8.0 claims, SD = 7.1), and patients in this group showed high medication claims, particularly for iron-chelating therapies (mean = 8.3, SD = 8.7), opioids (mean = 7.4, SD = 7.3), and hydroxyurea (mean = 5.0, SD = 3.1). This group had the highest numbers of opioid claims.

## Discussion

Results from the current study highlight the substantial burden of SCD affecting adolescents prior to the time in which they transition to adult care. The majority (~60%) of patients with SCD were affected by chronic complications during the one-year follow-up, with the most common complications being pulmonary. The overall patient cohort experienced an average of nearly 4 (SD = 3.9) VOCs, which demonstrates the substantial pain and healthcare burden of the disease. Further, the subset of patients with more VOCs requiring hospitalization had higher overall LOS and more opioid-related medication claims. Our results showed that SCD management for patients with SCD aged 16 to 18 years of age relies heavily on healthcare providers, blood transfusions, iron-chelating therapies, hydroxyurea, and opioids.

While many articles note the increase in hospital utilization in young adults, few studies have directly compared utilization patterns and treatments in adolescents to young adults.^23,35^ In our study, patients relied heavily on hydroxyurea and blood transfusions, which often require a hematologist or knowledgeable provider. Thus, it is necessary to ensure patients remain in the care of a specialist at the time of transition or the lack of these treatments will likely enhance health care utilization.

VOCs are a key reason patients with SCD seek medical attention.^36^ One study that included transition-aged SCD patients 16 to 25 years of age found that these patients experienced 32.7% of days with pain and 8.4% of VOC days.^22^ The mean pain intensity, on a scale from 1 to 9, was 4.2 on pain days; 5.6 on pain crisis days; 3.8 on non-VOC pain days. Roughly half (50.1%) of the home pain days were treated with opioids.^22^ About one third (36.7%) of ambulatory visits required care in the ER while nearly 1 in 5 (19.8%) crises were treated in the hospital rather than at home.^22^ The current study found comparable results with about 29.5% (5.4 of 18.3) of total HCRU visits were related to ER and hospital admissions suggesting these patients are already reliant on emergency care to manage their most painful episodes prior to transitioning to adult care. These data further confirm the high home opioid requirements of adolescents prior to transitioning to adult healthcare.

Similarly, in a large study of SCD patients (aged 1 to ≥65 years), Brousseau and colleagues found that young adult patients (18 to 30 years of age) had the highest healthcare utilization at 3.61 encounters per year.^35^ Our study reported 2 to 8 visits (including hospital, ER, office, and other outpatient visits) during the one-year follow-up in patients 16 to 18 years of age. The Brousseau study found children with SCD aged 10 to 17 years had 0.68 ER visits and 1.37 inpatient visits compared with 1.59 and 2.02, respectively, for those young adult patients aged 18 to 30 years.^35^ This may have changed as patients have aged. In this study, patients 16 to 18 years of age reported 3.4 ER visits and 2.0 inpatient visit in the overall analysis. These data further confirm that patients transitioning to adult care are already exhibiting high acute HCRU and require additional attention.

In patients with SCD, acute coronary syndrome (ACS) is a frequent cause and complication of hospitalization.^1,13,37,38^ In our study, chronic pulmonary disease was the most frequently observed complication and was recorded in about 1 in 3 (31.1%) patients in the overall cohort. It is likely that ACS was a component of the pulmonary disease results. The fact that so many of the patients in this study had pulmonary complications contributed to the HCRU findings (including hospitalization and outpatient visits). A published review of SCD showed that ACS occurs in about 15 to 40% of SCS patients, confirming the prevalence found in the current study.^1^ It is also well-known that ACS is the leading cause of death in adults with SCD. One study of nearly 25 000 hospitalization found an in-hospital mortality rate of 1.6%.^39^

For children and young adults with SCD, increased access to and utilization of healthcare services are key factors in decreasing morbidity and mortality.^32^ The Pain in Sickle Cell Epidemiology Study (PiSCES) demonstrated fewer physical challenges and pain frequency, HCRU, and better QoL, in transition-aged (16 to 25 years) versus older adults (37 to 64 years) with SCD.^22^ Another retrospective study found patients transitioning to adulthood relied more on emergency departments for their care than their pediatric counterparts.^10^ Raphael and colleagues found children with SCD 15 to 18 years of age had higher odds of high resource hospitalizations (odds ratio [OR] 3.39, 95% confidence interval 2.54–4.53) compared with children 0 to 4 years of age.^30^ Panepinto and associates found that for SCD patients ≤18 years of age, their LOS was longest in those 15 to 18 years of age (5.4 days).^31^ In a retrospective longitudinal cohort study, patients with SCD transitioning to adult care were found to receive fewer transfusions and hydroxyurea and fewer iron-chelating therapies.^11^ These results underscore the need for improved care during this period for this SCD patient group.

While life expectancy has been improving, patterns of morbidity and mortality show young adults particularly vulnerable to SCD-related early death.^14,40^ These young adults were at higher risk for death shortly after their transition to adult medical care.^15^ In pediatric care facilities, many children are managed by their hematology providers in the inpatient setting. In contrast, many adults living with SCD are managed by hospitalists, which may account for differences in outcomes related to SCD complications such as ACS.

Cerebrovascular complications for children and young adults with SCD have been evaluated previously.^41,42^ A retrospective study in SCD patients aged 0 to 18 years showed the incidence of hospitalization for stroke decreased across the US after the approval of hydroxyurea in 1998.^41^ The mean annual incidence rate of hospitalization from stroke decreased by nearly half (45%) from 0.51 per 100 patient years in 1993–1998 to 0.28 per 100 patient years in 1999–2009.^41^ While rates of ischemic stroke improved with age, the longitudinal Cooperative Study of Sickle Cell Disease found the incidence of hemorrhagic stroke increased with age and for patients 10 to 19 years of age with sickle cell anemia, the incidence of cerebrovascular accidents was 0.41/100 patient years.^42^ In the current study, cerebrovascular disease complications made up a substantial proportion (5.8%) of the complications in the overall patient cohort. It is important to continue to ensure assessment of these complications during the transition period.

## Limitations

The current study results are limited by the nature of database reviews. Widely varied definitions of SCD and complications across different studies exist in the literature.^1,4,12,18,20–23,26,28–31,35–37,39–43^ These data sets typically contain limited, de-identified information on hospitals, clinicians, and patients. These data include demographics and clinical information that is reliant on accurate recording of ICD and/or CPT diagnosis and procedure codes.^44^ Further secondary databases document paid claims for medications, but do not guarantee medications were actually taken. Only 16-18-year-olds were evaluated, and this study was descriptive in nature.

## Implications and Contribution

SCD places a significant burden on 16-to-18-year-old patients in terms of acute and chronic complications, HCRU, disease interventions, and medications prior to their transition to adult care. Attention to mental health, including pain coping and management, should be given to these high-risk patients prior to their transition to new providers and systems of care. Individuals with higher care needs require a provider with hematologic/SCD-specific. These results underscore the need for effective management strategies for SCD in this patient group prior to their transition to adult healthcare

**Source of Funding:** Novartis Pharmaceuticals Corporation.

## Disclosure of Potential Conflicts

Julie Kanter is the Director, Adult Sickle Cell Program, and Associate Professor of Medicine and Pediatrics at the Division of Hematology and Oncology in University of Alabama at Birmingham. Dr. Kanter is a consultant for Novartis and has served on scientific advisory boards for Novartis, bluebird bio, Imara, MODUS and also received honoraria from Medscape, Peervoice and Rockpointe for developing CME material.

Menaka Bhor, Frank (Yunfeng) Li, and Jincy Paulose were a full-time employee of Novartis Pharmaceuticals Corporation when this study was conducted.

Xin Li was an employee of KMK Consulting Service and worked at Novartis Pharmaceuticals Corporation as a contracted consultant when this study was conducted.

**Acknowledgments:** The authors would like to thank Write All, Inc. for editorial and writing services.

## Figures and Tables

**Figure:** Supplementary Content
